# Exploring Vitamin B12 Supplementation in the Vegan Population: A Scoping Review of the Evidence

**DOI:** 10.3390/nu16101442

**Published:** 2024-05-10

**Authors:** Sávio Fernandes, Leandro Oliveira, Alda Pereira, Maria do Céu Costa, António Raposo, Ariana Saraiva, Bruno Magalhães

**Affiliations:** 1 CBIOS (Research Center for Biosciences and Health Technologies), Universidade Lusófona de Humanidades e Tecnologias, Campo Grande 376, 1749-024 Lisboa, Portugal; saviof.nt@gmail.com (S.F.); leandroliveira.nut@gmail.com (L.O.); 2 Coimbra Health School, Polytechnic Institute of Coimbra, Rua 5 de Outubro—S. Martinho do Bispo, Apartado 7006, 3046-854 Coimbra, Portugal; 3 Institute for Preventive Medicine and Public Health, Faculty of Medicine, University of Lisbon, 1649-028 Lisboa, Portugal; aldapsilva@medicina.ulisboa.pt; 4 University Clinic of General and Family Medicine, Ecogenetics and Human Health Unity, Institute for Environmental Health, Instituto de Saúde Ambiental (ISAMB), 1649-028 Lisboa, Portugal; 5 Núcleo de Investigação em Ciências e Tecnologias da Saúde (NICiTeS), Polytechnic Institute of Lusophony, ERISA—Escola Superior de Saúde Ribeiro Sanches, 1900-693 Lisboa, Portugal; 6 Department of Animal Pathology and Production, Bromatology and Food Technology, Faculty of Veterinary, Universidad de Las Palmas de Gran Canaria, Trasmontaña s/n, 35413 Arucas, Spain; ariana_23@outlook.pt; 7 School of Health, University of Trás-os-Montes and Alto Douro (UTAD), 5000-801 Vila Real, Portugal; brunomm@utad.pt; 8 RISE—Health Research Network, Faculty of Medicine, University of Porto, 4099-002 Porto, Portugal; 9 Clinical Academic Centre of Trás-os-Montes and Alto Douro (CACTMAD), 5000-801 Vila Real, Portugal

**Keywords:** cobalamin, food supplements, plant-based diet, vegan diet, vegetarianism, vitamin B12

## Abstract

With a significant portion of the population adopting veganism and conflicting views among nutrition professionals regarding the necessity of vitamin B12 supplementation, this review aims to explore existing studies evaluating interventions through food supplementation. It focuses on the impact of vitamin B12 deficiency across different demographics. The present study seeks to understand how research has addressed the relationship between the rise in veganism and vitamin B12 deficiency over the past decade. A scoping review was conducted following the PRISMA flow diagram. Studies from 2010 to 2023 were identified using Boolean operators and key terms in electronic databases such as PubMed/MEDLINE, Web of Science, and EBSCO (Library, Information Science & Technology Abstracts, and Academic Search Complete). Out of 217 articles identified, 70 studies were included. The topical analysis categorized the studies into three groups: those associating vitamin B12 deficiency with diseases (*n* = 14), those analyzing the dietary habits of vegetarian individuals (vegan or not) without a specific focus on vitamin B12 (*n* = 49), and those addressing food guides and nutrition institution positions (*n* = 7). The authors concluded that vitamin B12 deficiency is prevalent among vegans due to limited consumption of animal products. For vegetarians, supplementation is an efficient means of treating and preventing deficiency; a daily dose of 50 to 100 micrograms is advised. There are still significant gaps in the research, nevertheless, such as the absence of randomized controlled trials evaluating various forms or dosages of vitamin B12 among vegetarians and the requirement for more information and awareness of the vitamin’s significance in vegan diets.

## 1. Introduction

Considering rapid population growth and inevitable pressures on the global food supply, such as the projected 44% increase in demand for animal products by 2050 to meet current global consumption levels, plant-based diets present a potentially healthier and more sustainable alternative [1].

“Vegetarian” is a term that defines a person who abstains from eating meat, fish, shellfish, and products made from these foods, optionally including other animal derivatives such as dairy, eggs, and honey in their diet [2,3]. Vegans, on the other hand, exclude any type of food or derivative of animal origin from their diet [2,3]. Choosing vegetarianism goes beyond food selection and reflects a philosophy aimed at reducing animal exploitation and cruelty, especially in food production. Additionally, this practice may be motivated by potential benefits for both health and the environment, making it an appealing option for many people [4,5]. Vegetarian diets are associated with a reduced risk of cardiovascular diseases by lowering modifiable risk factors like abdominal obesity, blood pressure, serum lipid profile, and blood glucose levels [6]. Additionally, these diets reduce inflammation markers, decrease oxidative stress, and protect against atherosclerotic plaque development, resulting in a lower risk of ischemic heart disease development and mortality [3,6].

Vegetarian diets are deemed beneficial for health, as they encourage diversity and stability of intestinal microbiota. This microbial diversity is significantly associated with body mass index (BMI), obesity, and cardiovascular protection [7]. Scientifically robust evidence suggests that vegan or vegetarian diets are linked to reductions in weight and BMI, and in certain instances, alterations in fat mass distribution [8].

In fact, well-designed vegetarian diets, including vegan diets, are considered healthy and nutritionally adequate, and may offer health advantages in preventing and managing specific disease risks. However, plant-based foods naturally lack a reliable source of vitamin B12, requiring individuals following a vegan diet to incorporate fortified foods or take vitamin B12 supplements to meet their dietary needs [9]. Inadequate vegetarian diets may lead to deficiencies in iron, calcium, zinc, vitamin D, and B12, as well as some amino acids. In some cases, this can result in hyperhomocysteinemia, protein deficiency, anemia, and decreased muscle creatinine [6,10].

The European Food Safety Authority (EFSA) proposes utilizing biomarkers such as serum cobalamin, holotranscobalamin, methylmalonic acid, and plasma total homocysteine, to establish Dietary Reference Values for cobalamin [11]. Despite uncertainties in defining cutoff values for vitamin B12 insufficiency and limited data, consistent evidence indicates that a daily intake of vitamin B12 at 4 µg/day or higher in adults is associated with adequate vitamin B12 status. Consequently, EFSA recommends an adequate intake (AI) of 4 µg/day for vitamin B12 in the European population, taking into account various biomarkers and observed intakes ranging from 4.2 to 8.6 µg/day in several EU countries [11].

Vitamin B12 deficiency varies depending on both the severity and the cause, being classified as mild, moderate, or severe, depending on measurable pathophysiological factors. The deficiency classified as mild generally arises as a result of inadequate intake, mainly among the differentiated population of vegans, vegetarians, individuals with low cobalamin consumption, and breastfeeding mothers with vitamin B12 deficiency. The body absorbs 1–5 mg of vitamin B12 daily and stores it substantially in the liver. However, noticeable clinical symptoms of deficiency arise only when vitamin levels fall significantly below the required limit. Determining serum vitamin B12 concentration is the main test to assess vitamin B12 status and risk of deficiency [12].

Some studies point to a deficient concentration of vitamin B12 (<156 pmol/L) as having a prevalence of 52% in vegan individuals and only 1% in omnivorous individuals [13]. Subnormal vitamin B12 status is prevalent (50–70%) in vegetarians or vegans in Austria, Germany, Italy, Australia, India, and China [14].

In this context, this scoping review aims to identify current studies concerning interventions through dietary supplementation of vitamin B12. It seeks to examine how this topic has evolved in recent years to address the genuine necessity for individual supplementation. Additionally, the present study aims to explore potential interventions tailored to specific vulnerable populations.

## 2. Methods

### 2.1. Study Design

To achieve the objectives, a scoping review of the scientific literature on vitamin B12 supplementation in vegan individuals was designed and the PRISMA model (Preferred Reporting Items for Systematic Reviews and Meta-Analyses) [15] was used to organize the information.

The formulation of the research question was based on the acronym PCC (population, context, and concept): What is known, from the existing literature, about vitamin B12 supplementation in vegan individuals?

The inclusion criteria for the articles are summarized in Table 1 and were defined based on the population, contexts and concepts, type of study, language, and date of publication.

The first methodological approach to identifying publications consisted of an exploratory search in electronic databases using previously defined keywords. In the next stage, the most relevant articles were consulted, the main phrases and search keywords were identified, and the terms of the Boolean phrases were defined to systematically carry out the final search. The respective descriptors in English were identified using the MeSH terms identified in the PubMed/MEDLINE, Web of Science, and EBSCO databases (Library, Information Science & Technology Abstracts, and Academic Search Complete).

Combinations of descriptors/medical subject headings (MeSH), subject headings, and subject terms were then applied to each of the databases, implementing the Boolean method operators “OR” and “AND” and the “*” tool, which leveraged research by creating new variations of the same word (Table S1).

### 2.2. Eligibility, Exclusion Criteria, and Selection Process

For original studies on the need for vitamin B12 supplementation, the following eligibility criteria were adopted for the included studies:

- Observation studies of individuals with a vegan diet;
- Studies that contain in their results an explicit, positive or negative, recommendation or indication of vitamin B12 supplementation.

All studies in which participants were involved in other studies without the participation of a population with a restricted vegan diet were excluded, as well as all studies in which there was supplementation by means other than oral administration, studies focused only on the development of new supplements on the market, studies on animals and, finally, studies that had been published in languages other than Portuguese, English, or Spanish.

For the selection process, two independent reviewers took on this task, reading in full the content available on the rayyan.ai^®^ platform (https://www.rayyan.ai/, accessed on 10 January 2024), and after analysis, they determined the study to be adequate or not (included or excluded). If the two reviewers simultaneously opted for the inclusion of an article, it was selected; in the same way, if one of the reviewers excluded the study while the other chose to include it, it was automatically classified as “perhaps,” with a third reviewer appointed as responsible for arbitrating the final decision on inclusion or non-inclusion. None of the studies needed to be classified in this way, and it was not necessary to appoint a third reviewer.

### 2.3. Data Collection/Extraction Process

As the selected studies repeatedly presented results or developments that were only partially conclusive on the topic, the data considered necessary for the soundness of the discussion and conclusion of this review were taken from the original texts based on the expertise of the reviewers. Data were extracted regarding the direct association of the vegan diet with possible vitamin B12 deficiency and their respective recommendations for supplementation or not. This process was carried out by three reviewers, the first being responsible for collecting information from study groups 2 (G2) and 3 (G3), and the second reviewer responsible for group 1 (G1). The third reviewer oversaw the revision of the final text, including all groups.

The data researched and considered relevant to the study came from the question “Is vitamin B12 supplementation necessary in vegan individuals?” To elucidate the question, the parameters described in this section were established. The articles and studies should then have been clear in terms of whether they recommend vitamin supplementation at any age, for any gender, and without violating the ethical principles of exclusion.

### 2.4. Synthesis Method

The process used to determine the eligible studies was carried out jointly by two reviewers, analyzing the complete studies in general but especially the results obtained and the discussion about the recommendation of vitamin B12 supplementation. Studies were selected if they deliberately mentioned a positive or negative recommendation for supplementation. All selected studies have full text. After the eligibility selection, relevant data were removed from each study, such as the authors’ names, country where it was carried out, year of publication, objective, abstract, and scope of the study.

This scoping review did not collect data for meta-analysis, as it was deemed unnecessary for the authors and the reviewers.

## 3. Results

**Figure 1:**
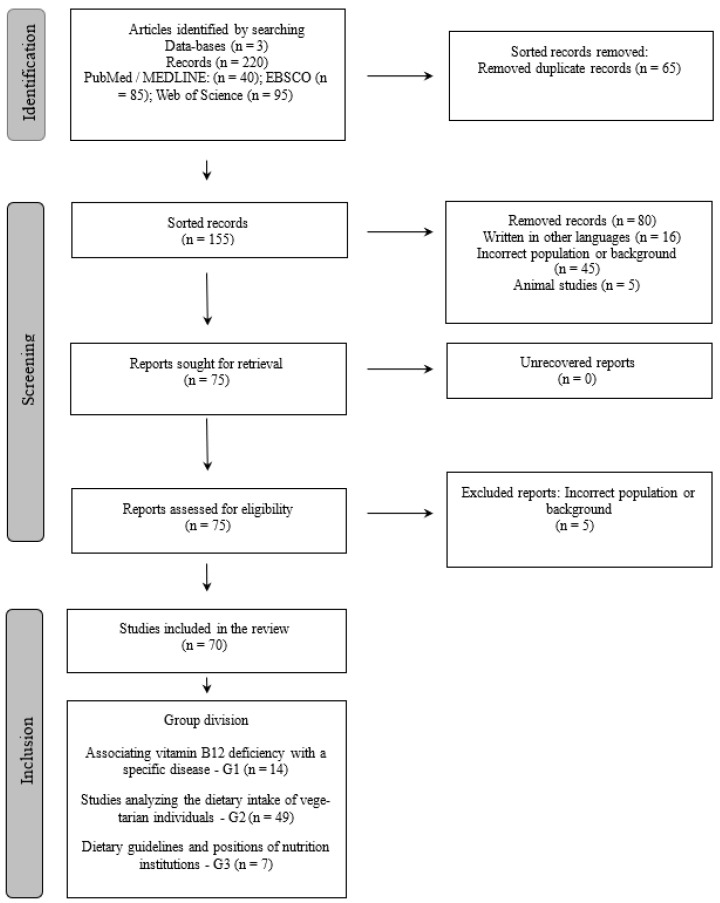
Process of identification and inclusion of articles—PRISMA diagram flowchart.

Considering the described procedures, the research results were refined, according to the previously established criteria and processes, until the final number of articles included in this review was reached. Figure 1 describes each one of the steps to reach the final number of articles included within the flowchart for the identification and selection of studies.

Using the outlined search strategy, a total of 218 articles were initially identified across various databases. Among these, 70 were found to be duplicates and thus were excluded. 

The remaining bibliographic sample consisted of 156 articles. However, upon closer examination, 15 articles were in languages other than the primary language of the review, 42 were deemed irrelevant due to incorrect population or background, and 5 articles focused on animal studies, which were deemed ethically discordant with the scope of this review. Additionally, 11 articles were related to specific commercial drugs, and 8 were rejected after a thorough reading of the full text. These findings were organized and summarized in Table 2, with the summaries prepared by the reviewers following a comprehensive analysis of the relevant information extracted from the full texts.

From the thematic analysis of all selected articles, three distinct groups of studies were defined as a posteriori by the researchers.

The first group consists of studies that focus on associating vitamin B12 deficiency with a disease (*n* = 14). A second group was formed by studies that analyzed the dietary intake of vegetarian individuals (vegan or not) without specifically focusing on vitamin B12 (*n* = 49). A third group was formed by studies on the composition of food guides and positions of nutrition institutions (*n* = 7).

### 3.1. Group 1—Association of Vitamin B12 Deficiency with Any Disease

#### 3.1.1. Illnesses in Infants

A vitamin B12 deficit in children is related to megaloblastic anemia from the mother or from a vegan and vegetarian diet without adequate supplementation [56,57]. The low level of this vitamin is only possible to transmit to the child when the mother exclusively breastfeeds until 6 months of age. In two studies, inappropriate levels of cobalamin were reported due to the type of diet of the vegan mother. One of the studies was about a 10-month-old child, and the other study related to a 6-month-old child. Both were female [56,57]. The children had identical symptoms: They could not stabilize their necks when sitting or grasping objects, and there was a regression in neurodevelopment and psychomotor activity [56,57].

Vitamin B12 deficiency in the first stage of life is rare, but it can be cured [57]. The treatment involves a scheme of cobalamin medicinal injections, the first stage of which consists of weekly injections for 4 weeks, then once a month for 3 months, and finally, once every 3 months for 6 months [57].

It is concluded that breastfeeding mothers who are vegetarian/vegan must pay special attention to their levels of vitamin B12 and take supplementation, if necessary, to avoid deficits of the same vitamin in infants.

#### 3.1.2. Childhood Illnesses

A recent study [54] had two objectives: (1) to find out the consequences of vegetarianism and veganism in children from conception to the end of the growth period and (2) to study the interaction potential of certain foods with physical and cognitive development. Through a controlled study design, where the target population was children who were vegetarian due to poverty, they studied whether milk, soy, and eggs would be equivalently good substitutes for meat. They divided the children into four groups: meat, milk, energy, and control. The first three groups were offered a dish based on products of vegetable origin with a supplementation of 60 g of meat, 200 mL of milk, or an extra 3 g of oil. In contrast, children who were part of the control group did not receive extra meals. Among the four study groups, the group in which there was an extra meat meal presented the best results, with improved physical activity during recess, an improvement in leadership activities, and a sense of initiative [54].

The relationship with acne was also investigated, for which diet is the crucial factor for its appearance. Acne is induced by hyperinsulinemia, and together with low insulin response and high protein doses, it has been deduced that these factors increase acne symptoms in child and adolescent patients [54].

Overall, this study concludes that a diet without food of animal origin or proper supplementation and care is neither beneficial nor safe for children’s health since it is associated with various risks to children’s growth, such as the risk of having a vitamin B12 deficiency, which can lead to delayed physical and cognitive development [54].

#### 3.1.3. Osteoporosis

Osteoporosis is recognized as a chronic disease that is associated with a progressive loss of bone mineral density, leading to a higher risk of fractures. To help combat or delay the development of this disease, calcium in the daily diet is foreseen as important, especially from foods such as milk and cheese. Vegetarian diets are poor in this mineral and in other vitamins such as cobalamin, since it is excluded from the diet. Nevertheless, it is determined that these diets are rich in other nutrients with a protective character, allowing vegetarians to have a low risk of bone fracture [33].

Osteoporosis risk increases with elevated serum methylmalonic acid levels, serving as a reliable indicator of insufficient vitamin B12 levels. Conversely, low vitamin B12 levels are linked to decreased mineral density, contributing to osteoporosis development [33].

Several studies have been carried out on vitamin B12 in association with low bone mineral density and consequently with osteoporosis. Studies in the target vegetarian population showed that a low concentration of cobalamin was associated with better bone remodeling and could lead to the acceleration of bone loss. In addition to this conclusion it was also discovered that a European study in which ovolactovegetarians, vegans, and omnivores were compared found that the prevalence of vitamin B12 deficiency was 77%, 92%, and 11%, respectively [33].

A vegetarian or vegan diet provides not only benefits for health but also risks, which can lead to malnutrition, that is, a deficit of certain nutrients such as vitamin B12, which can lead to neural or physical diseases such as osteoporosis and a higher risk of fractures [33]. However, if the individual is properly supplemented and followed by a professional, the health risk is lower and a healthy life may be achieved.

#### 3.1.4. Cardiovascular Diseases

The main reason a vegan diet is followed is the belief that it is better for health, as this type of diet has been reported as being potentially cardiac protective. The literature proves that there is a lower prevalence of hypercholesterolemia, hypertension, diabetes mellitus, and mortality due to heart attack among vegetarians [16]. As a rule, it is believed that vegetarian diets are protective of cardiovascular pathologies, although numerous studies prove the opposite [51]. The population following a vegetarian diet has a high prevalence of cobalamin deficiency, excessive salt consumption, and increased serum concentration of triglycerides, which may lead to certain pathologies such as arteriosclerosis [51]. To assess the impact of vitamin B12 supplementation, 50 vegetarian communities were studied, with an average of 14 years; non-smokers; no known kidney, vascular, or liver diseases; and no medication or supplementation taken [51]. A food frequency questionnaire was applied to estimate food intake over one week [51]. After applying this questionnaire, individuals were randomly assessed and received 500 µg of vitamin B12 orally per day for 12 weeks [51].

Research has shown that supplementing vitamin B12 in deficient vegetarians enhances arterial endothelial function compared to those receiving a placebo. Vegetarian diets, rich in plant-based compounds, are often linked with cardiovascular health benefits, including lower serum cholesterol levels and potentially reduced blood pressure [51]. However, cohort studies indicate that vegetarian diets may not significantly lower mortality rates from cardiovascular disease, particularly among strict vegetarians [51]. Notably, vitamin B12 deficiency is prevalent among two demographic groups: the elderly and vegetarians, often without noticeable symptoms. Nonetheless, a more advanced stage of cardiovascular disease can be prevented through vitamin B12 supplementation and adequate consumption of dairy products, eggs, and fortified cereals [51].

It is reasonable to assert that vegetarian diets offer protection for cardiovascular health. Moreover, vegan diets, when compared to lacto-ovo-vegetarian diets, demonstrate protective effects against high body mass index (BMI) and the prevalence of diabetes mellitus, hypertension, and hyperlipidemia [16]. It is also believed that vitamin B12 supplementation in vitamin B12-impaired but asymptomatic vegetarians may lead to a significant improvement in arterial endothelial function and carotid intimal thickness with possible potential to improve cardiovascular health [51]. Thus, one may conclude that the consumption of dairy products and eggs combined with the consumption of vegetables, fruits, cereals, and vitamin C can alleviate the adverse metabolic and vascular effects, resulting in a beneficial outcome for cardiac health [16].

#### 3.1.5. Delirium (Clinical Case)

Cobalamin deficiency affects about 20% of the elderly population and is considered a public health problem, and its symptoms can manifest at a neuropsychiatric, gastrointestinal, and hematological level, or in this case, symptoms such as depression, dementia, and delirium [62].

Cobalamin deficiency occurs due to poor absorption and pernicious anemia, making food play a fundamental role in preventing the problem. Therefore, it is necessary to pay attention to vegetarians as a risk group for contracting this type of condition [62]. Neuropsychiatric problems derive from the involvement of vitamin B12 with folate and homocysteine [62]. In this article, a case of a patient who contracted a psychological pathology due to cobalamin deficiency was studied [62].

The clinical case refers to a 62-year-old woman of Argentine origin who was admitted to the emergency room of a hospital. She was found lost on the street and showed signs of confusion. The patient complained that she suffered from insomnia, fatigue, and lack of concentration. After some tests, it was concluded that she had a psychomotor delay, anxiety, and depression [62].

This patient had a history of vitamin B12 deficiency but no reported neuropsychiatric symptoms potentially causing deficiency. The cause was found to be the vegetarian diet that the patient followed, which led to cases of delirium [62]. Weekly injections of 1000 μg of cyanocobalamin were applied to treat the patient. The levels of vitamin B12 normalized after a week and after 2 weeks. After psychiatric examination, the cognitive impairment had decreased as well as a part of the remission of the symptoms of depression [62]. Four weeks after the incident, tests showed that the patient was mentally stable and in complete remission of her depression symptoms [62].

### 3.2. Group 2—Studies Analyzing the Food Consumption of Vegetarian Individuals (Vegan or Not) without a Specific Focus on Vitamin B12

It has been demonstrated that vegans are deficient in vitamin B12, and it is generally agreed upon that this vitamin has to be supplemented [44]. Indicators of vitamin B12 deficiency (homocysteine, plasma methylmalonic acid, and holotranscobalamin II) show that vegans have lower levels of vitamin B12, but relatively few report clinical symptoms [34]. Compared with non-vegetarians, vegetarians have lower mortality from ischemic heart disease and lower BMI, serum glucose, serum cholesterol, and blood pressure [21]. Although long-term intake of a vegan diet is associated with these benefits, concentrations of essential nutrients are compared with baseline values [19]. Studies using plant-based foods to increase cobalamin intake are promising, but more data are needed [21]. The frequency of vitamin B12 deficiency is higher in people who do not take supplements or consume foods fortified with B12 than those who follow other vegetarian diets [20,27].

The vitamin B12 status of predominantly overweight or obese people is low, especially when they are vegetarians, as has been seen [58]. However, a well-planned vegetarian diet is healthy if nutritionally adequate and may help prevent and treat certain chronic diseases [31]. Since a vegan diet provides adequate nutritional recommendations for all nutrients except vitamins B12 and D, as well as calcium, in these cases, the use of fortified foods or food supplements becomes essential to ensure adequate nutritional support [61,76]. Dietary/food supplements containing vitamin B12 contribute significantly to plasma vitamin B12 levels, especially in vegans and ovolactovegetarians, followed by vitamin-fortified milk replacers in non-supplemented users. Milk replacer significantly impacts plasma vitamin B12 concentrations among individuals who are not receiving any type of supplementation [28]. This situation would be of particular importance for women of reproductive age to prevent the risks associated with maternal–fetal vitamin B12 deficiency [52].

Public health services, nutritionists, complementary and alternative therapists, and medical professionals must consider the needs of vegans, especially concerning monitoring cobalamin status to prevent cobalamin deficiency [53]. In Schüpbach et al. [14], it is noted that despite the relatively low consumption of vitamin B12 among vegans in Switzerland, deficiency rates remain low due to the prevalent use of dietary supplements. Likewise, Chandra-Hioe et al. [12] conducted a study in Australians seeking to understand the consumption of foods that provide adequate vitamin B12 for individuals on an omnivore diet, such as dairy products and meat. However, there seems to be a lack of knowledge about the consumption of foods such as vitamin B12-enriched foods and beverages by the general population, where vegan diets, even occasionally, are becoming more common. Data available from the Australian Health Survey on the consumption of fortified foods (such as soy milk) are limited to consumption by different age groups of the population and provide the only distinction between the sexes. This information can serve as a valuable resource in designing nutritionally balanced diets for both vegetarians and non-vegetarians. Additionally, it can inform the design of future clinical trials aimed at investigating the efficacy of various dietary sources of vitamin B12 in preventing deficiency among individuals who are not aware of the use of vitamin B12 supplements [28].

Regarding a more specific audience referring to puerperal women, infants, and children in early childhood, it was noted that vitamin B12 deficiency is a preventable cause of maternal and childhood diseases. As evidenced by Cruchet et al. [35], who sought to elucidate the myths and facts of vegetarian and gluten-free diets concerning infant feeding, vegetarianism does not represent any nutritional threat, as it normally includes egg and milk. Under expert supervision and with the appropriate vitamin B12 dosage, children get all the nutrients they need. However, vegan diets are not advised for everyone at any age, especially in vulnerable periods of life. If followed under the guidance of a professional, a vegetarian diet might be an effective alternative. Bandyopadhyay et al. [69] state that the UK NSC (National Safety Council) should consider including vitamin B12 deficiency in prenatal and neonatal screening in high-risk groups [69].

Mearns et al. [60] developed a semi-quantitative food frequency questionnaire (vitamin B12 FFQ) that is a non-invasive, easy-to-administer tool with moderate predictive ability to screen inadequate dietary vitamin B12 intake in South Asian women. Identifying women of childbearing age at low risk of vitamin B12 deficiency due to inadequate dietary intake offers the opportunity to offer dietary advice to prevent vitamin B12 deficiency or, where appropriate, to intervene with low-dose oral vitamin B12 supplements to treat early depletion or vitamin B12 deficiency. This is a significant public health announcement that can help lower the dangers to the health of mothers and their unborn children that come with a mother’s lack of vitamin B12 during pregnancy and lactation. It is especially relevant to communities in South Asia.

Chouraqui et al. [67] investigated possible associations of religious dietary rules, including strict vegetarian diets, with potential nutritional consequences. The conclusion was that, when implemented according to prescribed rules, most religious dietary precepts may be classified as not harmful to health, as evidenced by their historical adherence over millennia. However, some practices can lead to nutritional inadequacies, leading to vitamin B12 deficiencies. Patients with low socioeconomic status, the child population, and women of reproductive age are at particular risk of such deficiencies.

Another important point observed was the different forms of presentation of vitamin B12 in the food supplement market. The most common forms are cyanocobalamin (cyanCbl) and methylcobalamin (methylCbl). Manufacturers advise using methylCbl, as it is a ready-to-use form of the vitamin, whereas cyanCbl needs to be activated before being used in metabolism. The lowest amounts of holotranscobalamin were seen in vegans who attempted to supplement with other foods (seaweed, kombucha, and other fermented items), consistently falling short of the necessary intake. Hence, it is recommended that vegan individuals should be instructed about vitamin B12 supplementation, the pharmaceutical forms available on the market and their actions, and choosing the ideal plan to avoid the appearance of vitamin B12 deficiency [71].

Finally, Costantino et al. [47] investigated dietary guides from different countries to establish an opinion pattern to compose a Spanish guide for the vegetarian population. This led to the conclusion that vegetarians differ in their dietary group composition and position. Every image featured stressed the need to consume grains, vegetables, fruits, legumes, soy products, and nuts daily. Even after reviewing 11 guides, it was concluded that there is no consensus regarding the needs regarding amounts and portions of vita-min B12 supplementation. Karlsen et al. [42] investigated the nutritional content of meals based on common dietary guidelines, such as the MyPlate program from the US Department of Agriculture, using the Nutrition Data System for Research (NDSR). They aimed to assess the food and nutrient levels of whole-food plant-based (WFPB) and vegan diets over a 30-day period, sourced from popular cookbooks and recipe websites. Their study also compared these findings to US dietary recommendations like dietary reference intakes (DRIs) and MyPlate meal guidelines. Using NDSR analyses, they found that WFPB diets differed significantly from MyPlate recommendations, providing a more nutrient-rich diet with lower amounts of refined grains and added sugars compared to typical American diets. However, they recommended supplementation with vitamins B12 and D [42].

### 3.3. Group 3—Studies on the Composition of Food Guides and Positions of Nutrition Institutions

The third category comprises publications from nutrition institutions, which encompass additional studies supplementing food guides and established stances regarding the necessity of vitamin B12 supplementation for vegetarians or vegans. These publications, including references [13,18,26,35,36,38,48], may delve into topics such as vitamin B12 deficiency among vegetarians and vegans, its causes and repercussions, factors influencing vitamin B12 absorption, and various methods of vitamin B12 supplementation. In addition, these studies could also discuss the available evidence on the use of vitamin B12 supplements in vegetarians and vegans and formulate alternatives to help nutrition and health professionals provide more accurate and scientifically supported information about the nutritional needs of vegetarians and vegans.

The EPIC-Oxford [18], the Oxford component of the European Prospective Investigation into Cancer and Nutrition (EPIC), verified the difference between omnivores, pescatarians, ovolactovegetarians, and restricted vegans, and it was observed that the last group has the lowest levels of vitamin B12, of which only 20.8% make use of vitamin B12 supplements, either on its own (exclusive supplements with the vitamin) or as a multivitamin, which do not contain sufficient amounts for adequate vitamin supplementation.

The Biomarkers of Nutrition for Development (BOND) Project study by Allen et al. [36] investigated the vitamin B12 state of the art. It was found that enough of the vitamin can be obtained through fortified foods such as breakfast cereals. Therefore, the Vegan Society in the UK recommends the consumption of various fortified foods in different meals of the day. Nutritional yeasts should not be considered fortified because they do not present sufficient amounts for supplementation [9]. In addition, infants of vegan mothers are at greater risk of developing vitamin deficiency, and only 50% of them take supplementation. With these facts, the Institute of Medicine (IOM) of the United States concluded that babies of vegan mothers should be supplemented with adequate intake (AI) of vitamin B12 starting at birth because their reserves will be low, as will the concentrations of vitamin B12 in breast milk. Within this context, another study by Rudloff et al. [38] carried out specifically in vegan children and adolescents indicated that this type of diet, when prolonged, can lead to vitamin deficiency. The type of vitamins added to fortified products should also be characterized, since the most recommended form is cyanocobalamin, which is more stable in this type of food [9].

In conclusion, vitamin B12 plays a crucial role in maintaining the normal functioning of the brain and nervous system, as well as in the formation of blood cells. The authors observed that insufficient intake of these nutrients among vegans can result in health issues such as fatigue, anemia, depression, and cognitive impairment. In addition, it is important to regularly monitor the serum amounts of vitamin B12 in the vegan population, and responsible professionals should always recommend supplementation when vitamin amounts are not reached with fortified foods alone [13,48].

## 4. Discussion

The data presented allow us to conclude that there are no long-term prospective studies evaluating the effect of a vegan diet on vitamin B12 status. To explore the evidence regarding the rise in veganism and its association with vitamin B12 deficiency across different demographics, we identified 70 studies. These included narrative reviews (24, 34.3%), cross-sectional analytical studies (25, 35.7%), institutional positions on vegetarian diets (4, 5.7%), systematic reviews (3, 4.3%), systematic reviews and meta-analyses (2, 2.9%), randomized controlled trials (2, 2.9%), cohort studies (2, 2.9%), case studies (5, 7.1%), practice guidelines (1, 1.4%), a randomized trial (1, 1.4%), and a randomized clinical trial (1, 1.4%).

These studies assessed interventions involving food supplementation, with consistently positive outcomes. Primarily, due to vitamin B12 being sourced from animal products, its deficiency is prevalent among vegans who do not consume animal products or who consume insufficient amounts of fortified foods containing the vitamin. For the general population, vitamin B12 is well recognized as an essential nutrient that plays a crucial role in DNA formation, the production of red blood cells, and the proper functioning of the nervous system. The EFSA Panel on Dietetic Products and Allergies [11] has established a cause-and-effect relationship between dietary vitamin B12 intake and several health benefits. These include the regulation of homocysteine metabolism, the promotion of normal neurological and psychological functions, and a reduction in fatigue and tiredness. Also recognized is the claim that it “supports folic acid metabolism, in succession: DNA synthesis” for the general population in terms of cell division. The consolidated list of health claims according to Article 13 of Regulation (EC) No. 1924/2006 [82] submitted by Member States contains other additional entry claims with corresponding conditions of use as follows:

○ Vitamin B12 contributes to normal energy-yielding metabolism;
○ Vitamin B12 contributes to the normal functioning of the nervous system;
○ Vitamin B12 contributes to normal homocysteine metabolism;
○ Vitamin B12 contributes to normal psychological function;
○ Vitamin B12 contributes to normal red blood cell formation;
○ Vitamin B12 contributes to the normal function of the immune system;
○ Vitamin B12 contributes to a reduction in tiredness and fatigue;
○ Vitamin B12 has a role in the process of cell division.

The Institute of Medicine (IoM) [83] and the EFSA Panel on Dietetic Products and Allergies [11] offer recommendations for vitamin B12 intake in healthy adults. The IoM suggests a daily intake of 2.4 micrograms of vitamin B12 for adults, while the EFSA recommends a daily intake of 4 micrograms for the same demographic. Regarding children, the IoM provides varying daily intake recommendations: 0.9 micrograms for ages 1 to 3 years, 1.2 micrograms for ages 4 to 8 years, and 1.8 micrograms for ages 9 to 13 years. Meanwhile, the EFSA advises daily intake ranges of 0.5 to 1.5 micrograms for ages 1 to 3 years, 1.0 to 2.0 micrograms for ages 4 to 8 years, and 2.0 to 4.0 micrograms for ages 9 to 13 years. Regarding pregnancy and lactation, the IoM recommends 2.6 micrograms of vitamin B12 daily for pregnant women and 2.8 micrograms for lactating women. In contrast, the EFSA suggests a higher daily intake of 4.5 micrograms for pregnant and breastfeeding women. It is relevant that daily intake recommendations may vary according to gender, age, physiological status, and other factors associated with individual health conditions. It is always recommended to consult a healthcare professional before starting any vitamin B12 supplementation or making dietary changes. 

Regularly monitoring blood levels is a crucial preventive step that everyone should undertake. This allows healthcare providers to assess whether supplementation or dosage adjustments are necessary. Common recommendations for preventive vitamin B12 supplementation in adults include taking a daily single dose of 50–100 µg [13,21], three daily doses of 2 µg [13], or a weekly dose of 2000 µg, either as a single intake or divided into two doses of 1000 µg each, taken twice a week [21].

A clinical study compared the effectiveness of taking a daily dose of 50 µg versus taking a single weekly dose of 2000 µg in improving vitamin B12 levels in vegetarians and vegans with mild deficiency. Surprisingly, no differences were observed between the groups, indicating that, despite higher doses potentially being associated with lower absorption, supplementation appears to be equally effective when the dose and frequency are appropriate, whether as a daily dose of 50 µg or a weekly dose of 2000 µg [84]. There is no recommended upper limit for vitamin B12 intake because it is a water-soluble vitamin, meaning that part of it is excreted in urine. Therefore, to date, no adverse effects associated with excessive supplement intake have been reported in healthy individuals [11].

Data on the cobalamin dose required to maintain normal hematological status and serum cobalamin levels in vegetarians have shown that daily losses of ca. 0.2% of body stores are possible irrespective of the size of the body pool. Those with pernicious anemia in remission are expected to have depleted stores and thus lower absolute daily losses than healthy individuals. Thus, an intake of 1.5–2 µg/day may be considered to represent a minimum requirement for the maintenance of normal hematological status, linked with low body stores of 1 to 2 mg. Higher body stores (2–3 mg) are currently observed in healthy individuals, whose maintenance would require higher concentrations of intake [11].

Also important is that there are inherited causes of deficiency (Imerslund–Gräsbeck syndrome) or acquired defects such as pernicious anemia because of problems of malabsorption or due to the impairment of transport of vitamin B12 within the body. Insufficient dietary intake is rare in adults living in developed countries but is more often reported in vegans [73]. Within this context, Bärebring et al. highlighted that no conclusions can be drawn on whether habitual B12 intake or an intake in line with guidelines is enough to prevent a deficiency status in vegetarians and vegans because of an absence of published prospective cohort studies relating to B12 intake to status among vegetarians and vegans [85].

Key concepts/definitions are well clarified in the literature: There are two known reactions in humans that need vitamin B12 as a coenzyme. One is the conversion of succinyl-CoA from methylmalonyl-coenzyme A (CoA) by methylmalonyl CoA mutase in mitochondria during propionate metabolism. The other is homocysteine cytosolic trans-methylation by 5-methyl-tetrahydrofolate (5-methyl-THF) to methionine synthase-produced methionine [86,87]. Vitamin B12 and folate interact in the latter reaction. Insufficient levels of both vitamins hinder the synthesis of methionine and its derivative S-adenosyl-methionine (SAM), leading to significant disruptions in normal cellular function. Methionine, an essential amino acid, relies heavily on recycling through the remethylation pathway for its various metabolic functions. SAM is the universal methyl donor in over 100 transmethylation reactions involving neurotransmitter nucleotide, amino acid, and phospholipid metabolism, as well as detoxification reactions. Tetrahydrofolate (THF), the fully reduced form of folate, is another product of the methionine synthase reaction [88]. Case studies on infants from mothers with undetected pernicious anemia or adhering to strict veganism indicate that clinical symptoms of deficiency may appear in infants at around four to seven months of age [89].

When examining how research has been conducted on this topic, we recall the EFSA’s recommendations [11] for researchers to pursue studies on vitamin B12 biomarkers as a function of habitual intake in adults, children, and infants, including during pregnancy and lactation, comparing vegans with other populations. Also, outcomes are still scarce for vegan populations, and further investigation is needed into the relationships between vitamin B12 intake, biomarkers, and health. Data should be gathered systematically through national programs on the bioavailability of vitamin B12 from various foods and dietary intake patterns about age and physiological states (e.g., pregnancy, lactation), namely, for vegetarians.

We have focused the aim of this review on looking for key characteristics or factors related to existing studies that evaluated interventions through food supplementation, whether the result was favorable or not, in the different target audiences, with a focus on vitamin B12 and the consequent effects of the deficiency of this essential nutrient. In this respect, the present review has identified considerable knowledge gaps.

This scoping review demonstrates several strengths. We rigorously adhered to best practices, such as prospectively registering the protocol, and followed the PRISMA statement for reporting systematic reviews and meta-analyses. Additionally, the review benefited from systematic execution by three independent reviewers and the inclusion of a diverse range of article types, facilitating comprehensive data synthesis. While robust in many aspects, this scoping review also presents several limitations. Firstly, there is a possibility of publication bias, wherein unpublished studies or those not available in the languages covered by the review might have been missed, potentially skewing the results. Secondly, despite our systematic approach, there is always a risk of selection bias, where certain studies might have been inadvertently overlooked or chosen with bias. Thirdly, the included studies exhibit heterogeneity in terms of design, methodology, and population, which could impact the synthesis of findings and the general applicability of the results. Additionally, the quality of the included studies varies, potentially influencing the reliability of our conclusions. Furthermore, some studies may lack sufficient data, limiting the depth of analysis and the certainty of the conclusions drawn. Lastly, there is a limitation regarding the time frame, as the review might not capture the most recent evidence due to constraints on literature search cutoff dates.

Future research endeavors should aim to fill these knowledge gaps, thereby offering improved guidance. The management of vitamin B12 deficiency in vegans is imperative, necessitating educational efforts, a general heightened awareness, and regular monitoring. Additionally, careful consideration of potential risks linked to excessive vitamin B12 supplementation is crucial in further studies. Furthermore, there is a pressing need for enhanced public education regarding the significance of vitamin B12 in vegan diets. This could entail the implementation of campaigns and outreach initiatives aimed at advocating for a balanced vegan diet that fulfills all nutritional needs.

## 5. Conclusions

This scoping review sheds light on the relationship between veganism and vitamin B12 deficiency. It aimed to find out how dietary supplementation affects different groups regarding vitamin B12 deficiency. The findings reveal that vitamin B12 deficiency is common among vegans due to their limited intake of animal products. The review identified 70 relevant studies, including various types such as reviews, trials, and guidelines. There are commercially available sources of vitamin B12 for vegans, such as fortified cereals, plant-based drinks, and nutritional yeast. Supplementation has been shown to effectively prevent and treat vitamin B12 deficiency in vegetarians, with daily doses ranging from 50 to 100 micrograms being sufficient. It is noteworthy that data on the dose of cobalamin required to maintain normal hematological status and serum cobalamin concentrations in vegetarian populations or individuals with low cobalamin intake have also been considered by some specialized bodies, but systematic data collection is lacking regarding cobalamin, with few references concerning the criteria (endpoints) on which dietary reference values are based, namely, indicators of the need for cobalamin to maintain a balanced hematological status. Healthcare professionals must educate vegan patients on monitoring and supplementing their vitamin B12 levels to prevent developmental delays. However, there are gaps in the research, such as the lack of randomized trials on vitamin B12 doses and the long-term effects of vegetarianism on health.

## Figures and Tables

**Table 1:** Definition of inclusion criteria.

	Inclusion Criteria
Population (P)	Studies in adults aged 18 years and older on a vegan diet
Context (C)	Studies in which participants receive oral vitamin B12 supplementation
Concept (C)	Studies that address the effects of vitamin B12
Types of Studies	All primary or secondary, qualitative or quantitative studies
Language	Studies published in English, Spanish, or Portuguese
Publication date	Studies published between January 2010 and December 2023

**Table 2:** Studies selected for the literature review.

**Study**	**Objectives**	**Type of Study**
E1 [16]	Associating atherosclerosis and the occurrence of vitamin B12 inadequacy	Randomized controlled trial
E2 [17]	Comparing the amounts of vitamin B12 and homocysteine in vegetarians and omnivores	Systematic review and meta-analysis
E3 [18]	Investigating the food intake of groups of 30–90-year-old carnivores, fish eaters, vegetarians, and vegans using semiquantitative diet frequency questionnaires	Analytical cross-sectional study
E4 [19]	Comparing standards of dietary intake and nutritional status of Finnish long-term vegans and non-vegetarians	Analytical cross-sectional study
E5 [20]	Examining prevalence values of cobalamin deficiency among vegetarian individuals assessed for serum vitamin B12	Systematic review
E6 [21]	Discussing vitamin B12 status of vegetarians with a focus both on the detection of cobalamin deficiency and appropriate sources for sufficient intake	Narrative review
E7 [22]	Guiding as to recommended nutrient (folate, vitamin B12, and vitamin B6) intake during pregnancy	Narrative review
E8 [23]	Using vitamin B12 and MMA markers to show the cobalamin status of Spanish vegetarians, along with the results of a plant-based diet and vitamin B12 supplementation	Analytical cross-sectional study
E9 [24]	Determining the impact of maternal diets on the levels of vitamin B12, ferritin, hemoglobin, and folic acid in mother’s blood and umbilical cord blood	Analytical cross-sectional study
E10 [25]	Analyzing breast milk vitamin B12 concentration and vitamin B12 supplement use patterns among women who adhered to vegan, vegetarian, and nonvegetarian different dietary patterns	Analytical cross-sectional study
E11 [26]	Establishing practical parameters of micronutrient consumption for children involved with vegan diets	Practice guidelines
E12 [27]	Analyzing serum status of folate and vitamin B12 in vegan and ovo-lacto vegetarians	Analytical cross-sectional study
E13 [28]	Investigating any association between the plasma concentration of vitamin B12 and the use of animal-based meals, fortified foods, and supplements by non-vegetarians and vegetarians	Analytical cross-sectional study
E14 [29]	Reviewing literature to provide recommendations for how to construct a vegan diet for athletes and exercisers	Narrative review
E15 [30]	Outlining the metabolism of vitamin B12 and assessing the causes and effects of subclinical B12 insufficiency	Narrative review
E16 [31]	Discussing the nutrients potentially related to major concerns in a vegetarian diet along with the health benefits of following a vegetarian diet	Narrative review
E17 [32]	Obtaining insights on the benefits and risks of vegetarianism, with emphasis on vegetarian child nutrition profiles	Narrative review
E18 [33]	Potential nutritional deficiencies can be avoided by carefully choosing meals, fortifying foods, or using supplements to assist in maintaining good bone condition and lower the risk of fracture in those who follow vegetarian diets	Narrative review
E19 [34]	Determining any extent to which the reason for following a vegan diet has been associated with healthy behaviors	Analytical cross-sectional study
E20 [9]	Position of the Academy of Nutrition and Dietetics about several aspects of vegetarian diets	Institution position
E21 [35]	Clarifying the myths and facts of vegetarian and gluten-free diets, according to the existing evidence	Narrative review
E22 [14]	Evaluating the overall intake and the status on selected vitamins and minerals among vegetarians and vegan adults living in Switzerland	Analytical cross-sectional study
E23 [13]	Position of the Working Group of the Italian Society of Human Nutrition about several aspects of vegetarian diets	Institution position
E24 [36]	Reporting on vitamin B-12 (B12) as part of the Biomarkers of Nutrition for Development (BOND) Project, providing advice and state of the art on the selection, use, and interpretation of biomarkers for nutrient exposure, function, and status, and also including a section on vegetarian diets	Narrative review
E25 [37]	Analyzing the nutritional sustainability of the vegetarian diet of professional dancers	Narrative review
E26 [38]	Position of the Working Group of the Nutrition Committee, German Society for Pediatric and Adolescent Medicine (DGKJ) about vegetarian diets in childhood and adolescence	Institution position
E27 [39]	Developing vegan meal plans that focus on nutrients commonly lacking in the diet for children	Analytical cross-sectional study
E28 [40]	Tracing the profile of consumed micronutrients and related biomarkers for vegetarian and vegan athletes	Cohort study
E29 [41]	Exploring the vitamin B12 status of toddlers living in high-poverty areas of China and observing the effects of different complementary foods on the cognitive level of these toddlers and vitamin B12 status	Analytical cross-sectional study
E30 [42]	Creating theoretical plant-based, whole-food meal plans that are vegan and based on popular nutrition and cookery sources that were found through a thorough web questionnaire	Analytical cross-sectional study
E31 [43]	Comparing the nutritional consumption of recreational runners who are omnivores, lacto-ovo vegetarians, and vegans with the intake guidelines recommended for the general public by the Austrian, German, and Swiss Nutrition Societies	Cohort study
E32 [44]	Comparing macro- and micronutrient availability among vegetarian and traditional healthy diets	Narrative review
E33 [45]	Summarizing existing knowledge on the variability of defined nutrients in the breastmilk of mothers who reported to adhere to a plant-based diet	Systematic review
E34 [12]	Determining the status of cobalamin between Australian vegetarians and vegans	Narrative review
E35 [46]	Presenting advantages and disadvantages of vitamin supplementation and the indications for it in diverse life situations	Narrative review
E36 [47]	Examining the primary visual representations of food-based vegetarian diet recommendations from many nations in order to develop a new guide tailored to this particular Spanish demographic	Narrative review
E37 [48]	Position of the Committee on Nutrition and Breastfeeding in The Spanish Paediatric Association on vegetarian diets in infants and children	Institution position
E38 [49]	Systematic review of the evidence of associations between seasonal affective disorder and diet, eating behavior, and nutrition intervention	Systematic review
E39 [50]	Evaluation of vitamin B12 status of apparently healthy Indian children 6–23 months of age	Analytical cross-sectional study
E40 [51]	Studying the impact in vegetarians of vitamin B12 supplementation on arterial function	Randomized study
E41 [52]	Identifying the prevalence of vitamin B12 deficiency in vegetarian Indians	Epidemiological study
E42 [53]	Identifying the prevalence of vitamin B12 deficiency in vegans who do not use supplements in the Czech Republic	Epidemiological study
E43 [54]	Determining whether a vegetarian diet is healthy for children	Narrative review
E44 [55]	Reviewing literature to provide recommendations for how to construct a vegan diet for special populations of athletes and exercisers	Narrative review
E45 [56]	Presenting data on the influence of maternal vitamin B12 deficiency in infant psychomotor retardation	Case study
E46 [57]	Reporting a case of a 10-month-old infant with developmental regression secondary to vitamin B12 deficiency	Case study
E47 [58]	Describing the vitamin B12 status of South Asian women residing in Auckland who are primarily overweight or obese and linking the relationship between insulin resistance and blood vitamin B12 and vegetarian status	Epidemiological study
E48 [59]	Concentrating information necessary for the successful planning of vegetarian food introduction in children	Narrative review
E49 [60]	Estimating dietary B12 intake in the population of women originating from South Asia	Epidemiological study
E50 [61]	Verifying if the vegan diet exempt from food of animal origin still supplies the nutritional recommendations of supporters	Narrative review
E51 [62]	Reporting on a patient whose etiological and diagnostic work-up revealed delirium caused by a vitamin B12 deficiency	Case study
E52 [63]	Reviewing literature to indicate the influence of mineral and vitamin supplements on pregnancy outcome	Narrative review
E53 [64]	Examining the effect of vitamin B deficiency on the adult nervous system and other clinical manifestations of vitamin B deficiency	Narrative review
E54 [65]	Examining and contrasting the health and demographic traits of Australian women of reproductive age who follow a vegan diet with those of the general population, finding sources and amounts of vitamin B12 and comparing them to current guidelines, and looking for correlations between participant attributes and sufficient vitamin B12 intake	Epidemiological study
E55 [66]	Analyzing the existing literature on the growth and health impact of selected nutrients in the vegan child population	Narrative review
E56 [67]	Reviewing the literature dealing with potential associations between religious food rules and potential nutritional outcomes	Narrative review
E57 [68]	Reviewing the literature on the association between pregnancy in vegan and vegetarian populations and vitamin B12 deficiency	Narrative review
E58 [69]	Examining the prevalence and typical forms of vitamin B12-deficient presentations among non-vegetarian patients aged 20 to 80 who visited a tertiary care hospital in the state of Eastern India	Analytical cross-sectional study
E59 [70]	Analyzing a population of women during early pregnancy in Nepal for cobalamin and folate status	Randomized controlled trial
E60 [71]	Analyzing the impact of two forms of vitamin B12 supplements (methylcobalamin and cyanocobalamin) on the amount of active blood vitamin (holotranscobalamin) in a group of Romanians who ate a plant-based, vegan diet	Analytical cross-sectional study
E61 [72]	Identifying if vitamin B12 insufficiency in newborns is caused by dietary issues or an inherited defect in absorption and metabolism	Case study
E62 [73]	Describing the role of vitamin B12 deficiency in cardiovascular disease development among vegetarians	Systematic review and meta analysis
E63 [74]	Reviewing the literature on issues of pregnancy in vegans	Narrative review
E64 [75]	Investigating the vegan diet as a cause of severe megaloblastic anemia and psychosis	Case study
E65 [76]	Estimating the impact of a vegan diet over time on blood B12 concentrations in healthy omnivorous individuals by contrasting the effects of eating natural products with those enriched with B12	Prospective analytical cross-sectional study
E66 [77]	Assessing the adequacy of vitamin B12 supplementation in Australian vegan study participants by comparing intake to RDI, outlining the range of supplementation habits, and commenting on appropriate supplementation regimens	Analytical cross-sectional study
E67 [78]	Finding out how common cobalamin insufficiency is and how often vegetarian and vegan children in the Czech Republic use vitamin B12 supplements	Analytical cross-sectional study
E68 [79]	Evaluating the nutritional status of healthy, young, and physically active individuals who followed different types of diets (omnivores, vegetarians, and vegans) in terms of various nutrients, including vitamin B12	Analytical cross-sectional study
E69 [80]	Identifying the prevalence of regular and irregular vitamin B12 supplementation in Slovak and Czech vegans	Analytical cross-sectional study
E70 [81]	Investigating health and supplementation behavior on vitamin B12 supplementation, and demographically characterizing the community of Austrian adult vegans. Additional aim: to evaluate adherence to check-ups and vitamin B12 supplementation among Austrian vegans, and the prevalence of healthy behaviors	Analytical cross-sectional study

## Supplementary Materials

The following supporting information can be downloaded at: https://www.mdpi.com/article/10.3390/nu16101442/s1, Table S1: Indexers used to select publications. Used Boolean phrases.
