# Supplementary material for: Exploring Vitamin B12 Supplementation in the Vegan Population: A Scoping Review of the Evidence
Source: Nutrients. 2024 May 10;16(10):1442. doi: 10.3390/nu16101442 (PMC11124153; doi:10.3390/nu16101442)
Supplement: Supplementary file 1 [file nutrients-16-01442-s001.zip › nutrients-2968086-supplementary.pdf]

nutrients MDPI

Supplementary Materials

Table S1. Used Boolean phrases.

**PubMed/Medline**	(((Dietary Supplement[MeSH Terms]) OR (Dietary Supplements[MeSH Terms]) OR (Dietary Supplementations[MeSH Terms]) OR (Food Supplements[MeSH Terms]) OR ("Food Supplementation") OR (Food Supplementations[MeSH Terms]) OR (Neutraceutical[MeSH Terms]) OR (Neutraceuticals[MeSH Terms]) OR (Nutraceutical[MeSH Terms]) OR (Nutraceuticals[MeSH Terms]) OR (Nutriceutical[MeSH Terms])) AND ((B12 vitaminamin[MeSH Terms]) OR (B 12 vitaminamin[MeSH Terms]) OR (Cobalamin[MeSH Terms]) OR (Cobalamins[MeSH Terms]) OR (Cyanocobalamin[MeSH Terms]) OR (vitaminamin B12 Deficiency[MeSH Terms]) OR (Deficiency, vitaminamin B12[MeSH Terms]) OR (Deficiencies, vitaminamin B12[MeSH Terms]) OR (vitaminamin B12 Deficiencies[MeSH Terms]) OR (Deficiency, vitaminamin B 12[MeSH Terms])) AND ((Vegan Diet[MeSH Terms]) OR (Vegan Diets[MeSH Terms]) OR (Veganism[MeSH Terms]) OR (Vegan[MeSH Terms]) OR (Vegetarian[MeSH Terms]) OR (Vegetarian Diet[MeSH Terms]) OR (Plant Based Diet[MeSH Terms]) OR (Plant Based Nutrition[MeSH Terms]) OR (Plant Based Diets[MeSH Terms]) OR (Vegetarianism[MeSH Terms])))
**Web of Science**	(((Dietary Supplement) OR (Dietary Supplements) OR (Dietary Supplementations) OR (Food Supplements) OR (Food Supplementation) OR (Food Supplementations) OR (Neutraceutical) OR (Neutraceuticals) OR (Nutraceutical) OR (Nutraceuticals) OR (Nutriceutical)) AND ((B12 vitaminamin) OR (B 12 vitaminamin) OR (Cobalamin) OR (Cobalamins) OR (Cyanocobalamin) OR (vitaminamin B12 Deficiency) OR (Deficiency, vitaminamin B12) OR (Deficiencies, vitaminamin B12) OR (vitaminamin B12 Deficiencies) OR (Deficiency, vitaminamin B 12)) AND ((Vegan Diet) OR (Vegan Diets) OR (Veganism) OR (Vegan) OR (Vegetarian) OR (Vegetarian Diet) OR (Plant Based Diet) OR (Plant Based Nutrition) OR (Plant Based Diets) OR (Vegetarianism)))
**EBSCO (Library, Information Science & Technology Abstracts e Academic Search Complete)**	(((Dietary Supplement) OR (Dietary Supplements) OR (Dietary Supplementations) OR (Food Supplements) OR (Food Supplementation) OR (Food Supplementations) OR (Neutraceutical) OR (Neutraceuticals) OR (Nutraceutical) OR (Nutraceuticals) OR (Nutriceutical)) AND ((B12 vitaminamin) OR (B 12 vitaminamin) OR (Cobalamin) OR (Cobalamins) OR (Cyanocobalamin) OR (vitaminamin B12 Deficiency) OR (Deficiency, vitaminamin B12) OR (Deficiencies, vitaminamin B12) OR (vitaminamin B12 Deficiencies) OR (Deficiency, vitaminamin B 12)) AND ((Vegan Diet) OR (Vegan Diets) OR (Veganism) OR (Vegan) OR (Vegetarian) OR (Vegetarian Diet) OR (Plant Based Diet) OR (Plant Based Nutrition) OR (Plant Based Diets) OR (Vegetarianism)))
